# Cold-endoscopic submucosal dissection: time to go further?

**DOI:** 10.1055/a-2700-9850

**Published:** 2025-11-27

**Authors:** Miguel Fraile-López, Alvaro Terán, Maria Luisa Cagigal, Adolfo Parra-Blanco

**Affiliations:** 1 Gastroenterology and Hepatology Department, Clinical and Translational Research in Digestive Diseases, Valdecilla Research Institute (IDIVAL), Marqués de Valdecilla University Hospital, Santander, Spain; 2 Pathology Department, Marqués de Valdecilla University Hospital, Santander, Spain; 3 9820 NIHR Nottingham Biomedical Research Centre, Nottingham University Hospitals NHS Trust and the University of Nottingham, Nottingham, United Kingdom

Endoscopic submucosal dissection (ESD) has become increasingly implemented in Western countries over the past decade to treat early neoplasia. However, its long learning curve and potential associated adverse events limit its routine use, particularly in challenging locations such as the colon. One of the main limitations of colonic ESD is the risk of intraprocedural and delayed perforation, due to the extremely thin muscular layer of the colon 1 .

Cold-ESD is a novel technique that minimizes the use of electrocautery, thereby reducing the risk of complications related to thermal injury. A 68-year-old woman underwent a screening colonoscopy observing a 30-mm flat elevated serrated lesion (0–IIa) with a 12-mm dysplastic area located in the transverse colon. Magnifying endoscopy revealed a JNET2B lesion with Kudo’s IIIL pit pattern, so “en bloc” resection was proposed. The C-ESD technique consists of submucosal injection with a mixed solution of gelafundin, indigo carmine, and adrenaline (1:500,000), followed by mucosal incision with multiple stepwise bites using a biopsy forceps (Radial Jaw 4; Boston Scientific, France), as previously described by our group 2 3 . After flap creation, early traction with a clip-band was applied to facilitate the identification of the dissection plane 4 5 . Once within the submucosa, dissection was carried out using a scissor-type knife (Clutch-Cutter DP2618DT; Fujifilm Medical, Tokyo, Japan) and an endoscopic Maryland dissector (Coag Dissector, Ovesco, Tübingen, Germany). These rotatable ESD knives enable blunt dissection of the submucosa by opening and closing the clamp while also allowing for coagulation of large vessels ( Video 1 , Fig. 1 ). Histological examination confirmed a serrated lesion with high-grade dysplasia and clear margins ( Fig. 2 ).

Cold-endoscopic submucosal dissection for a flat elevated serrated lesion with dysplasia in the transverse colon.Video 1

**Fig. 1:**
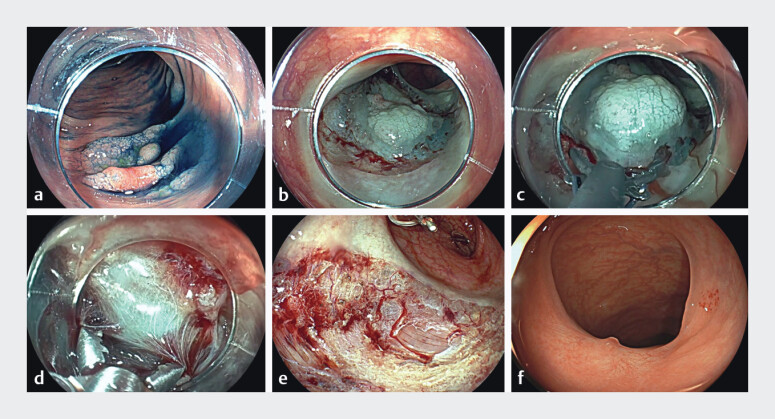
30-mm flat elevated (0–IIa) serrated lesion with a 12-mm dysplastic area in transverse colon. **a** Indigo carmine highlights the morphology. **b** Circumferential incision was completed with biopsy forceps. **c, d** Blunt dissection was performed using a scissor-type knife and Maryland dissector while opening and closing the clamp and selective coagulations of vessels. **e** Cold-ESD scar with no thermal damage to the muscular layer. **f** Twelve months’ follow-up with complete healing.

**Fig. 2:**
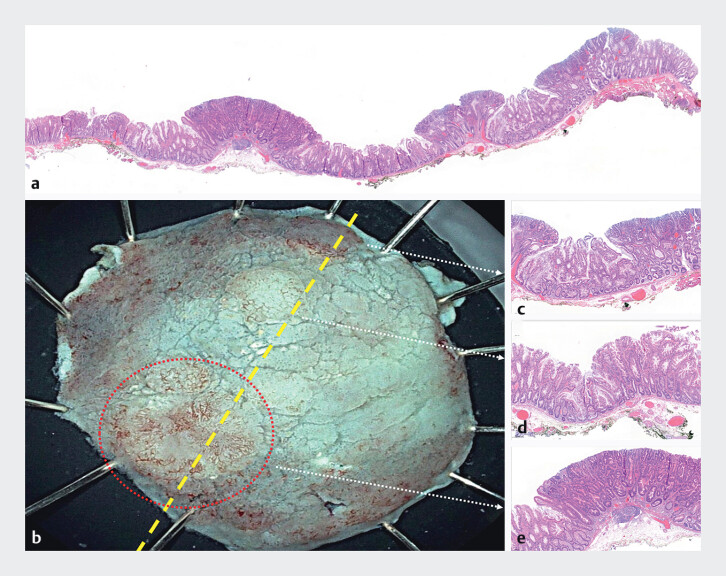
**a** Histological section of a sessile serrated lesion with three dysplastic lesions (hematoxylin–eosin [HE] 2×). **b** Cold-ESD specimen with the yellow line representing the previous histological section. **c** Sessile serrated lesion with low-grade dysplasia (HE: 2×). **d** Sessile serrated lesion without dysplasia (HE: 2×). **e** Sessile serrated lesion with high-grade dysplasia (HE: 2×).

Cold-ESD reduces the risks associated with electrocautery, which could improve the safety of ESD in difficult locations such as the colon. The development of specific endoscopic devices tailored for this technique will be crucial for its wider implementation.

Endoscopy_UCTN_Code_TTT_1AQ_2AD_3AD

Citation Format

Endoscopy 2025; 57: E752–E753. DOI: 10.1055/a-2626-3707

## Notes

Endoscopy E-Videos https://eref.thieme.de/e-videos
*E-Videos* is an open access online section of the journal *Endoscopy* , reporting on interesting cases and new techniques in gastroenterological endoscopy. All papers include a high-quality video and are published with a Creative Commons CC-BY license. Endoscopy E-Videos qualify for HINARI discounts and waivers and eligibility is automatically checked during the submission process. We grant 100% waivers to articles whose corresponding authors are based in Group A countries and 50% waivers to those who are based in Group B countries as classified by Research4Life (see: https://www.research4life.org/access/eligibility/ ). This section has its own submission website at https://mc.manuscriptcentral.com/e-videos .
